# Parachute Technique: A New Endoscopic Method for Closing Recurrent Oronasal Fistulas in Cleft Palate Patients

**DOI:** 10.3390/jcm14124299

**Published:** 2025-06-17

**Authors:** Aleksander Zwierz, Oskar Komisarek, Paweł Burduk

**Affiliations:** Department of Otolaryngology, Audiology and Phoniatrics, Collegium Medicum, Nicolaus Copernicus University in Torun, Jagiellońska 13-15, 85-067 Bydgoszcz, Poland; aleksanderzwierz@gmail.com (A.Z.); pburduk@cm.umk.pl (P.B.)

**Keywords:** oronasal fistula, cleft palate, parachute technique, endoscopic repair, inferior nasal turbinate flap

## Abstract

**Objective**: To present an innovative endoscopic method, the “Parachute Technique,” for effectively closing recurrent oronasal fistulas in cleft palate patients using autologous tissue. **Summary Background Data**: Oronasal fistulas are common complications after cleft palate repair, often leading to impaired quality of life due to difficulties with speech, eating, and an increased risk of infections. Current surgical methods exhibit high recurrence rates, especially in cases involving significant scarring or large defects. Therefore, there is a need for new techniques that improve outcomes and reduce recurrence. **Methods**: The study introduced the “Parachute Technique,” which uses autologous tissue from the inferior nasal turbinate to create a mucosal flap. This flap is transposed through the fistula using a guidewire under endoscopic guidance. The endoscopic approach minimized trauma to the surrounding tissues and allowed for precise manipulation during the procedure. **Results**: The “Parachute Technique” successfully closed recurrent oronasal fistulas, particularly in cases where conventional surgical methods had failed. The use of autologous tissue reduced the immunological risks, while the minimally invasive nature of the endoscopic procedure decreased the postoperative morbidity and improved the healing outcomes. **Conclusions**: The “Parachute Technique” offers a promising alternative for the treatment of recurrent oronasal fistulas in cleft palate patients, providing a minimally invasive, effective solution that can be easily adopted by specialists across multiple surgical disciplines.

## 1. Introduction

Oronasal fistulas are common complications following primary cleft palate repair, significantly affecting patients’ quality of life by impairing speech and eating functions and increasing the risk of respiratory infections [1]. The development of these fistulas results from a combination of anatomical and procedural factors. The primary causes of fistula formation include the type and extent of the cleft, surgical technique and operator experience, and biological factors.

Patients with complete cleft palates, classified as Veau classifications III and IV, and those with concurrent cleft lip are more prone to fistula development [2]. A wider cleft increases the tension at the closure site, potentially leading to wound dehiscence [3].

Additionally, the choice of closure method and the surgeon’s skill are crucial [4]. Less experienced surgeons may inadequately assess the tissue tension or improperly prepare flaps, increasing the risk of complications [5]. Biological factors such as insufficient vascularization of tissues, the presence of scars from previous surgeries, and infections can adversely affect the healing process, promoting fistula formation [6].

Various surgical methods are employed to close oronasal fistulas. Local mucoperiosteal flaps utilize adjacent tissues to cover the defect. While suitable for smaller fistulas, they may have a high recurrence rate in cases of large defects or scarred tissues [7]. Tongue flaps involve the use of well-vascularized flaps from the tongue to close large fistulas. Although effective, they can cause patient discomfort and require a longer healing period [8]. Tissue grafts, such as fascia lata or dermal fat grafts, allow for the closure of defects when local tissues are insufficient [9]. Alloplastic materials and biomaterials, including collagen membranes, titanium meshes, or bone substitutes, can support tissue regeneration and provide additional structural support [10]. Microvascular and endoscopic techniques represent modern methods that enable the precise closure of fistulas with minimal invasiveness; an example is endoscope-assisted repair using a graft from the inferior turbinate [11].

Despite the availability of numerous methods, the recurrence rate of fistulas remains significant, reaching up to 68% in some adult patient groups [1]. Factors contributing to recurrences include tension at the closure site, insufficient vascularization, the presence of scars and previous surgeries, and postoperative infections [12]. Excessive tension can lead to wound edge separation, while poor blood supply limits the healing capacity [13]. Scar tissue can hinder tissue mobilization and affect elasticity, and a wound infection can cause dehiscence and the formation of new fistulas [14].

There is a clear need for developing new, more effective methods for closing oronasal fistulas that minimize the risk of recurrence and improve both the functional and aesthetic outcomes. In response to these challenges, we present an innovative endoscopic technique for closing recurrent oronasal fistulas in cleft patients termed the “Parachute Technique”. The aim of this method is to enhance the treatment efficacy through a novel surgical approach that minimizes invasiveness and supports natural healing processes.

## 2. Procedure Description

**Figure 1:**
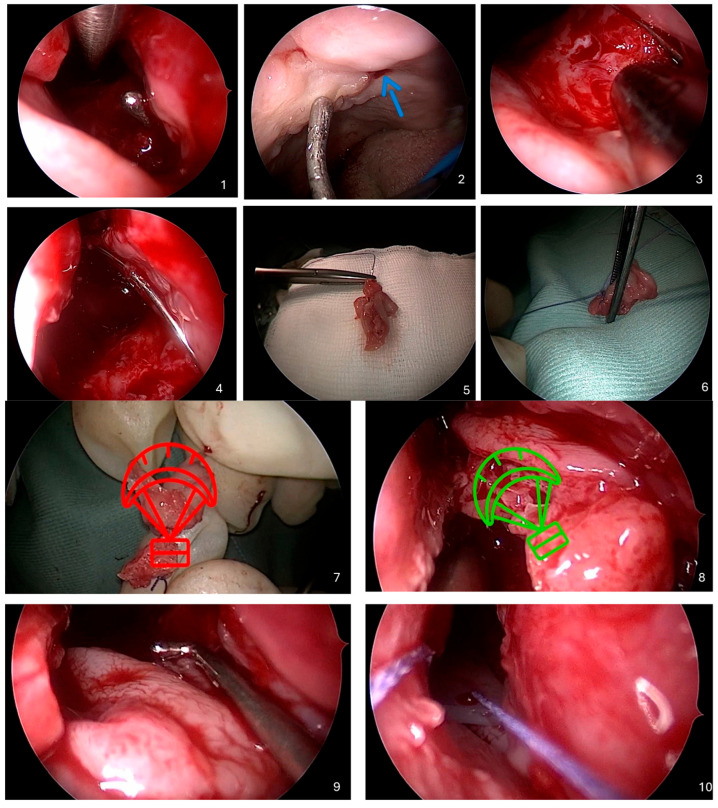
A step-by-step illustration of the “Parachute Technique” for closing a recurrent oronasal fistula in patients with cleft palate, comprising ten sequential images: (**1**,**2**) Identification of the oronasal fistula: The introduction of a sterile probe through the oral cavity into the oronasal fistula to precisely determine its location and size. Simultaneously, an endoscope is inserted into the nasal cavity to visualize the tip of the probe protruding through the fistula from both sides. (**3**) Preparation of the nasal cavity: Thorough cleaning of the area around the fistula in the nasal cavity using saline irrigation to remove mucus, debris, and any crusts. Granulation or scar tissue around the edges of the fistula is gently removed under endoscopic control. (**4**) Passing the needle: Insertion of a sterile needle through the fistula from the oral cavity into the nasal cavity using a guidewire. The needle is advanced through the nasal cavity and exited through the anterior nostril. (**5**–**7**) Preparation of the flap from the inferior nasal turbinate: The identification and excision of the inferior nasal turbinate under endoscopic guidance. The excised turbinate is prepared by partially separating the mucous membrane from its bony part without complete detachment, allowing it to be unfolded into a “parachute” shape. The needle is passed through the excised turbinate, piercing it to half its thickness. (**8**,**9**) Placement and securing of the flap: The needle is carefully withdrawn back through the anterior nostril, nasal cavity, and fistula into the oral cavity, pulling the prepared flap along with it. Under endoscopic control, the flap is positioned to provide full and even coverage of the fistula edges, with the mucosal surface facing the nasal cavity. The flap is secured in place and a guide suture is fixed to the oral cavity. (**10**) Application of a silicone dressing: A thin layer of medical-grade silicone or a silicone stent is applied to the nasal side of the flap to protect it, stabilize it, and prevent the formation of adhesions in the nasal cavity.

Below is a detailed description of the Parachute Technique, an endoscopic method developed for closing recurrent oronasal fistulas in cleft palate patients. A video recording of the entire procedure is provided, and each step is illustrated with intraoperative figures (Figure 1).

1. Identification of the Oronasal Fistula

The procedure begins by inserting a sterile probe through the oral cavity into the oronasal fistula to precisely determine its location and size. Simultaneously, a 0-degree or 30-degree rigid endoscope is introduced into the nasal cavity. Using the endoscope, the surgeon locates the tip of the probe protruding through the fistula, allowing visualization of the defect from both sides.

2. Preparation of the Nasal Cavity

The area around the fistula in the nasal cavity is thoroughly cleaned using saline irrigation to remove mucus, debris, and any crusts. Granulation or scar tissue around the edges of the fistula is gently removed using a microdebrider under endoscopic control. Local vasoconstrictive agents, such as adrenaline-soaked tampons, or electrocautery are applied to minimize bleeding during the procedure.

3. Excision of the Inferior Nasal Turbinate

Under endoscopic guidance, the inferior nasal turbinate on the side of the fistula is identified.

Optionally, the inferior turbinate on the contralateral side may be selected if it is larger and more suitable for reconstruction. Using microsurgical instruments, the inferior nasal turbinate is excised at its base, separating it from the lateral wall of the nasal cavity. The excised turbinate is carefully removed through the anterior nostril and placed on the operating table outside the nasal cavity for further preparation.

4. Preparation of the Flap from the Inferior Nasal Turbinate

Using a guidewire, a sterile needle is inserted through the fistula from the oral cavity into the nasal cavity. The needle is then advanced through the nasal cavity and exited through the anterior nostril. The needle is passed through the excised inferior nasal turbinate, piercing it to half its thickness. Without completely detaching it, the mucous membrane of the inferior nasal turbinate is gently partially separated from its bony part. This partial separation allows the mucous membrane to be unfolded, forming a “parachute” shape.

5. Transposition of the Flap Through the Fistula

The needle is carefully withdrawn back through the anterior nostril, nasal cavity, and fistula into the oral cavity, pulling the flap of the inferior nasal turbinate along with it. In this way, the flap is transposed through the fistula from the nasal cavity into the oral cavity. The surgeon ensures that the mucosal ‘parachute’ is properly positioned, with the mucosal surface facing the nasal cavity, while the bony parts of the inferior turbinate effectively plug the fistula channel.

6. Securing the Flap

Under endoscopic guidance, the flap position is carefully adjusted to ensure complete and uniform coverage of the fistula margins. Any folds or creases that could impede the healing process are meticulously avoided. The flap is anchored in place by fixing an absorbable guiding suture of the parachute flap to the mucosa of the oral cavity near the fistula’s ostium. Ensuring the flap is tension-free is essential for adequate vascularization and optimal healing.

7. Application of Silicone Dressing

A thin layer of medical-grade silicone or a silicone stent is placed on the nasal aspect of the flap. This dressing serves to protect and stabilize the flap while preventing adhesion formation within the nasal cavity. The silicone dressing is carefully shaped to conform to the nasal anatomy without obstructing the airflow and is anchored with nonabsorbable sutures at the base of the nasal vestibule. If necessary, gentle packing is also applied to further support the structure.

8. Postoperative Management

The patient is instructed to gently cleanse the nasal cavity with saline sprays or irrigations to maintain moisture and prevent crusting. A soft diet is recommended, and activities that could increase nasal pressure, such as nose blowing or strenuous physical exertion, should be avoided. Prophylactic antibiotics are prescribed to minimize the risk of infection, and analgesics are provided as needed for pain management. Regular follow-up appointments are scheduled to monitor the healing progress and evaluate the success of the procedure. The silicone stent is removed after 14 days, with endoscopic examinations performed as necessary.

### Remarks

The procedure is performed under general anesthesia with endotracheal intubation, ensuring patient safety and optimal surgical conditions. Strict aseptic conditions are maintained throughout the procedure to minimize the risk of infection. Gentle handling of the mucous membrane and bony tissue is crucial to preserve their integrity and viability. This technique is particularly useful for patients with recurrent fistulas where previous methods have failed and there is a need for an alternative approach.

## 3. Discussion

Closing recurrent oronasal fistulas in patients with cleft palate presents a significant surgical challenge due to scar tissue formation, the limited availability of healthy tissues, and a high risk of recurrence [9]. The introduction of the Parachute Technique offers a novel approach to addressing these difficult cases. This method involves utilizing the excised inferior nasal turbinate to create a mucosal flap shaped like a “parachute,” which is then transposed through the fistula using a guidewire and needle. By employing the patient’s own tissues, this technique allows for a multilayered closure of the defect, promoting better healing and integration with surrounding structures.

One of the primary advantages of the Parachute Technique is the use of autologous tissues. Employing the patient’s own mucosal and bony tissues eliminates the risk of immunological reactions and rejection, which can enhance the healing process and improve the long-term success of the repair [8]. Additionally, the technique is minimally invasive due to the use of endoscopic guidance. This allows for precise manipulation within a confined surgical field, minimizing damage to adjacent tissues and reducing postoperative morbidity [11].

The effectiveness of this method is particularly notable in difficult cases where traditional techniques have failed. Patients with recurrent fistulas, especially those complicated by scar tissue from previous surgeries, may benefit significantly from this approach. The technique avoids the need to harvest tissues from distant donor sites, thereby reducing additional trauma and potential complications in other areas of the body. Furthermore, the multilayered closure provided by the Parachute Technique promotes faster tissue regeneration and decreases the risk of residual fistula formation, potentially leading to improved patient outcomes. An additional advantage of the Parachute Technique is its role in preventing ascending infections of the nasal cavity and paranasal sinuses. By effectively sealing the oronasal communication, the technique limits the transfer of oral flora, food debris, and liquids into the nasal passages. This is particularly relevant in patients with a profoundly altered oral ecosystem, which is often observed in individuals with long-standing or recurrent fistulas. In such cases, chronic exposure of the nasal cavity to microbial and enzymatic agents originating from the oral environment significantly increases the risk of local inflammation and secondary infection. By restoring anatomical separation between the oral and nasal compartments, the technique helps re-establish the local microbiological balance and promotes long-term mucosal health.

However, several limitations and drawbacks of the Parachute Technique should be considered. This procedure demands advanced endoscopic skills and proficiency in instrument manipulation within a confined surgical field, requiring a level of expertise that may not be available in all centers, thereby potentially limiting its widespread adoption. Collaboration between specialists from various fields, such as maxillofacial surgery, plastic surgery, and ENT, could help address this challenge. Furthermore, the precision and delicacy required during the procedure can extend the operation time compared with traditional techniques, increasing the overall burden on both the patient and the surgical team.

Anatomical variations or previous surgical interventions may also pose challenges. In some patients, the inferior nasal turbinate may not be suitable for excision or use as a flap, limiting the applicability of the technique [15]. Additionally, although the inferior nasal turbinate is not considered a critical structure, its removal can lead to nasal function disorders, such as mucosal dryness, epistaxis, or alterations in nasal airflow patterns [16]. These potential complications necessitate careful patient selection and thorough preoperative evaluation.

The requirement for specialized equipment is another consideration. The necessity of using an endoscope and specific surgical instruments may present challenges in facilities with limited access to modern medical technology [14]. This factor could impede the implementation of the Parachute Technique in resource-constrained settings, where traditional methods might remain the standard of care.

In conclusion, the Parachute Technique represents a promising alternative for the treatment of recurrent oronasal fistulas in patients with a cleft palate. Its main advantages include the utilization of autologous tissues, minimal invasiveness, and effectiveness in challenging cases where other methods have failed. Nevertheless, to facilitate the broader adoption of this technique, it is essential to acknowledge and address its limitations. These include the need for advanced endoscopic skills, potential complications associated with the removal of the inferior nasal turbinate, and the requirement for specialized equipment. Ongoing research, along with targeted training programs for surgeons, may help optimize the technique and expand its applicability in clinical practice. By carefully weighing the benefits against the drawbacks, clinicians can make informed decisions about incorporating the Parachute Technique into their surgical repertoire for the management of recurrent oronasal fistulas.

## 4. Conclusions

In conclusion, the Parachute Technique offers a promising alternative for the treatment of recurrent oronasal fistulas in cleft palate patients. By addressing many of the challenges associated with traditional surgical methods, it aims to enhance the treatment efficacy, reduce the recurrence rates, and improve both the functional and aesthetic outcomes. With further research and training, this technique has the potential to become a valuable addition to clinical practice, contributing to better patient outcomes and satisfaction.

## Data Availability

No new data were created or analyzed in this study. Data sharing is not applicable to this article.
